# Evaluation of eczema, asthma, allergic rhinitis and allergies among the Grade-1 children of Iqaluit

**DOI:** 10.1186/s13223-018-0232-2

**Published:** 2018-02-27

**Authors:** Ahmed Ahmed, Amir Hakim, Allan Becker

**Affiliations:** 1 Department of Pediatrics, University of Ottawa, Ottawa, ON Canada; 2 National Heart and Lung Institute, Imperial College, London, UK; 3 Section of Allergy and Clinical Immunology, Department of Pediatrics and Child Health, University of Manitoba, Winnipeg, MB Canada

**Keywords:** Asthma, Allergic rhinitis, Eczema, Allergies, Inuit, Nunavut, Subarctic, Children

## Abstract

**Background:**

Little is known about the prevalence of asthma, allergic rhinitis, eczema and allergies among Canadian Inuit children, especially those living in the arctic and subarctic areas.

**Methods:**

A cross-sectional study among Grade 1 students attending schools in Iqaluit, the capital of Nunavut, was conducted during the 2015/2016 school year. We used the International Study of Allergy and Asthma in Children questionnaire with added questions relevant to the population. In addition, skin prick tests were conducted to test for sensitization to common food and environmental allergens.

**Results:**

The prevalence of current asthma was 15.9% (> 2:1 males) with the highest prevalence among those with any non-Inuit heritage at 38.5%. The prevalence of current and past allergic rhinitis was 6.8%, also predominant among males, with the lowest prevalence among the mixed ethnicity. Home crowdedness was inversely related to past asthma. Being ever outside Nunavut was associated with higher prevalence of current and past asthma. No statistically significant relationship was found with passive smoking or exclusive breast feeding during the first 4 months of life. The current eczema prevalence was 20.5%, with the highest prevalence recorded among the Inuit at 25% compared to 15.4% among the mixed ethnicity and 14.3% among the non-Inuit. We noted a high rate of sensitization to cat at 26.7% while absent sensitization to other common inhalant allergens.

**Conclusion:**

Variations in the prevalence and risk factors of asthma, allergic rhinitis and eczema among different ethnicities living at the same subarctic environment may be related to genetic, gene-environment interaction and/or lifestyle factors that require further investigation.

**Electronic supplementary material:**

The online version of this article (10.1186/s13223-018-0232-2) contains supplementary material, which is available to authorized users.

## Background

Little is known about the prevalence of asthma, allergic rhinitis, eczema and allergies among the Canadian Inuit children, especially those living in the Canadian territory of Nunavut. This is the first study addressing that issue among Grade 1 students in Nunavut. Improving our knowledge of those conditions among the Inuit children carries the potential of improving their prevention and management.

**Fig. 1:**
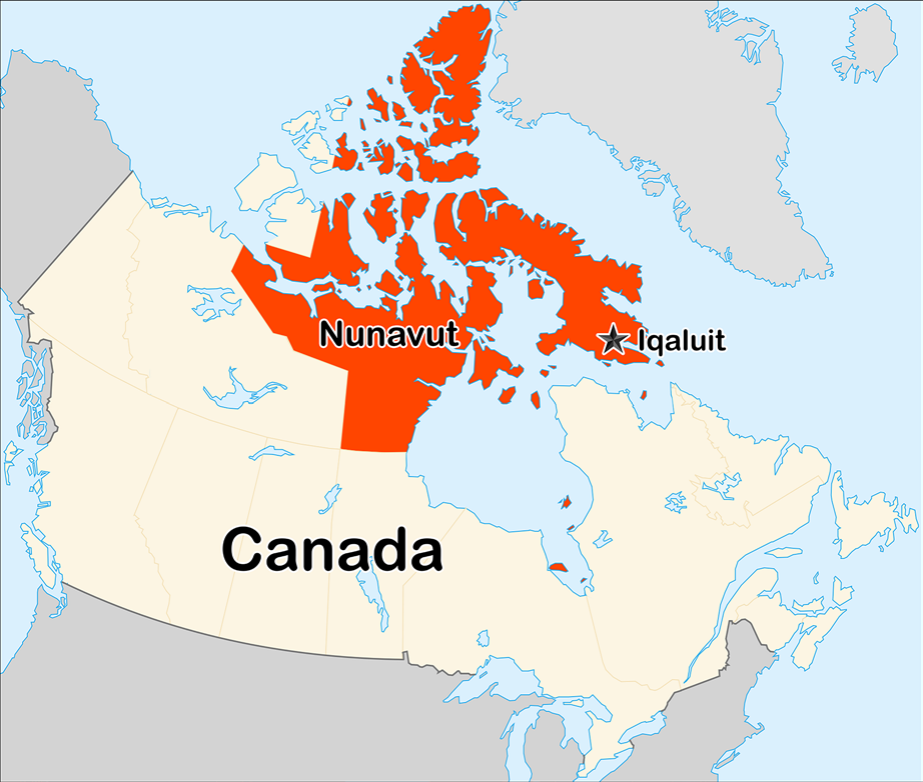
Maps of Nunavut

Nunavut is a sparsely populated area of arctic and subarctic tundra located above latitude 60° with a surface area of over 2 million sq. km, (800,000 sq. miles) and a population of 36,702 as of January 1, 2015, the vast majority being of indigenous Inuit heritage. Huge areas of its surface are sheathed in ice year-round, having more than 50% of Nunavut’s landmass above the Arctic Circle with not a single tree in the entire area. The City of Iqaluit is the capital of Nunavut being the largest community with a population of around 7250. Forty-one percent of the population is under the age of 16. Iqaluit is located at latitude 63°77′North and longitude 68°54′West (Fig. 1). Winters can be very harsh with average temperatures of − 27 °C in Iqaluit which limits the time of out-door activities for most of the year [1–4].

The International Study of Asthma and Allergies in Childhood (ISAAC) has shown marked variations in the prevalence of atopic diseases between countries and within countries over time [5, 6]. A number of studies have also reported a higher prevalence of allergy and eczema in colder, more northern regions [7–11].

There is a wide variation in the prevalence of asthma in Canada as found by Hong-Yu Wang et al. with the highest prevalence of asthma in Halifax at 33%, was more than double the lowest found in Vancouver at 13.7% [12]. The ISAAC study has never been conducted in Nunavut. Forsey found an eczema rate of 16.5% among Inuit children (age 2–12 years) in the Labrador area (a province south eastern to Nunavut with an Inuit minority). Two-thirds of these children presented with moderate or severe eczema, with a high female/male ratio of 2:1. Food specific IgE antibody assays showed that 32, 23, and 5% of Inuit children with eczema were sensitized to egg, milk, and wheat, respectively, while none of the controls were sensitized [13].

Asthma and allergies are among the most common chronic conditions reported by parents/guardians of indigenous children under the age of 6 years [14, 15]. Chang et al. found that children and adults with Inuit ancestry, but living outside Nunavut and off the First Nations reserves in other provinces, had a significantly lower prevalence of asthma and allergies compared to children from other indigenous groups [16].

In a study of Inuit school children in northern Quebec (a Canadian province southern to Nunavut), specific sensitization to dust mite was very unusual with almost complete absence of mite allergen in house dust (none of 50 dust samples taken from the mattress or bedroom floor contained 1 mg or more of allergen per gram of dust) [17]. Alaskan native children residing in rural Alaska have a low prevalence of allergic sensitization to inhalant allergens [18].

There is an increased burden of Tuberculosis (TB) among children in Nunavut, where over 12% of their reported cases were pediatric cases, compared to 7% for Canada [19]. All children born in Nunavut are eligible to receive the Bacille Calmette–Guérin (BCG) vaccine at birth. Many studies have tried to determine whether a relationship exists between TB infection, BCG vaccine and the prevalence of atopic diseases but reported inconsistent findings [20–23].

This study was part of a bigger research project (evaluation of eczema, asthma and allergies among the children of Iqaluit; EAACI). It also investigated if there is any discrepancy between the Inuit and non-Inuit children living in the same harsh environment as well as any possible relation to certain risk/protecting factors.

## Methods

The city of Iqaluit was chosen because it is the most populated city in Nunavut so that a larger cohort can be studied. The study was approved by the Research Ethics Board at the University of Manitoba and received permission from the Nunavut Research Institute.

### Study design

The study was conducted between November 2015 and February 2016 at the Qikiqtani General Hospital (Iqaluit). It is cross-sectional with the study population being all Grade 1 students attending the four elementary schools at Iqaluit during the academic year 2015/2016, a total of one hundred and thirty students, with no exclusion criteria. The families were contacted multiple times over 2 months through the schools by delivery of the study invitation and questionnaire to the students and over the phone by the study assistants. Because of major concerns expressed related to the issue of research conducted during the era of residential schools and clearly un-ethical aspects of that research, we were not allowed any other form of recruitment. The study included two components; a questionnaire and skin prick testing.

### The questionnaire

A questionnaire of 30 questions (see Additional file 1: Appendix S1) adopted with modification from the ISAAC study with additional questions relevant to the Nunavut population including locally applicable risk factors (see Additional file 2: Appendix S2). The consent form and assent form were available in English and Inuktitut languages, all the parents were satisfied to use the English one. Two study coordinators were hired, one is a local Inuit that speaks Inuktitut, to minimize the language barrier bias, and however, most of the Inuit in Iqaluit are fluent in English.

### The skin prick test

The skin testing was performed to 14 common allergens (food and inhalant), see Additional file 3: Appendix S3. It was performed and interpreted by Dr. Ahmed Ahmed at the Qikiqtani General Hospital outpatient clinic over 2 days in February 2016. The skin prick epicutaneous testing was performed to a variety of common food and inhalant allergens with positive histamine and negative saline controls. The allergen extracts and the testing devices were products of Lincoln Diagnostics, New York, USA (ALK-Abello Pharmaceuticals, Inc, Mississauga, Ontario, Canada).

The food extracts included common six food allergens: cow’s milk, soy, egg white, wheat, peanut and tree nut mix (equal portions of almond, Brazil nut, pecan nut and pistachio). The answered questionnaires did not have evidence for fish or shellfish allergy and these were not included in the food panel. The environmental inhalant allergens included tree mix (nine equal parts of Alder, Ash, Elm, Birch, Maple, Hickory, Oak, Poplar and Sycamore trees), Grass mix [mixture of five standardized grass pollens: Timothy (*Phleum pratense*), Orchard (*Dactylis glomerata*), June (*Poa pratensis*), Redtop (*Agrostis alba*) and Sweet Vernal (*Anthoxanthum odoratum*)], Ragweed mix (two equal parts of short and tall ragweed), Weed mix (four equal parts of Rough, Pigweed, English plantain and Lamb’s quarters), Mold mix (four equal parts of Alternaria, Sphaerospermum, Mixed-Aspergillus and Mixed-Penicillium), House dust mite (HDM) (two equal parts of Dermatophagoides pteronyssinus and Dermatophagoides farinae), cat (standardized cat pelt) and dog Epithelium. Histamine (10 mg/mL) and saline solution (0.9%) were used as positive and negative controls, respectively. On the day of testing, the skin prick testing method and consent forms were reviewed with the parent and the child. In addition, an assent form was discussed with the child with either a verbal or written assent confirmed.

The skin prick test was epicutaneous and read at 15–20 min by Dr. Ahmed. A wheal with a mean diameter of at least 3 mm greater than the saline control was considered positive. There were no negative histamine control or any positive saline control.

### Data analysis

The statistical analyses was performed using IBM SPSS v.22 (BM Corp. Released 2013. IBM SPSS Statistics for Windows, Version 22.0. Armonk, NY: IBM Corp.)

Data were analyzed using correlations, cross tabulations (Chi square; Fisher’s exact test), and cross tabulations with risk analyses (odds ratios, 95% confidence interval).

## Results

There were 130 Grade 1 students in Iqaluit at the time of the study. Forty-four families (33.8%) provided consent for the child to be enrolled in the study (all of them agreed to participate in both parts of the study; the questionnaire and the skin prick test) but only thirty children (23.1% of the total cohort) attended the skin prick testing despite a reminder call a day earlier.

### Study demographics

The ethnic distribution of participants who completed the questionnaire (44 cases) was as follows: Inuit 54.5%, non-Inuit 15.9% and mixed ethnicity (one of the parents is Inuit) 29.5%. Of those who attended the skin prick test, 56.7% were Inuit, 20% non-Inuit and 23.3% of the mixed ethnicity. There were 26 males and 18 females.

### Asthma prevalence

Following a standard ISAAC approach, based on the ISAAC questionnaire primary question, the prevalence of current asthma was 15.9% and the male to female ratio was 2.5:1 (Table 1). The prevalence of current asthma in this population was highest among those children of mixed ethnicity at 38.5% and lowest among the Inuit at 4.2% with prevalence amongst non-Inuit of 14.3%, this difference among ethnicities was statistically significant (Fisher’s exact test 6.798 and p = 0.016)

**Table 1:** Summary of the findings in relation to current and past asthma

The questionnaire was completed by 44 subjects	Current asthma	Past asthma
Male:female Odds ratio 95% CI	5:2 1.91 [0.33, 11.12]	7:4 1.29 [0.32, 5.28]
Ethnicity Inuit, n (%) Mixed, n (%) Non-Inuit, n (%) Total, n (%) Chi square (p-value)	1 (4.2) 5 (38.5) 1 (14.3) 7 (15.9) 6.80 (0.02)	4 (16.7) 6 (46.2) 1 (14.3) 11 (25.0) 3.96 (0.15)
Smoker in the home (yes:no) Odds ratio 95% CI	3:4 1.39 [0.27, 7.15]	4:7 1.0 [0.24, 4.13]
Crowdedness correlation (p)	0.165 (0.29)	0.285 (0.06)
Cat owner (yes:no) Odds ratio 95% CI	1:3 3.0 [0.20, 44.36]	1:5 1.60 [0.12, 21.59]
Dog owner (yes:no) Odds ratio 95% CI	2:3 1.0 [0.15, 6.91]	4:5 1.28 [0.28, 5.93]
Exclusive breast feeding (yes:no) Odds ratio 95% CI	4:3 0.81 [0.16, 4.18]	5:6 0.42 [0.10, 1.67]
Being ever outside Nunavut (yes:no) Odds ratio 95% CI	7:0 n/a n/a	11:00 n/a n/a
Previous respiratory hospitalization (yes:no) Odds ratio 95% CI	2:5 1.71 [0.27, 10.74]	3:8 1.69 [0.34, 8.31]
TB vaccination (yes:no) Odds ratio 95% CI	6:0 n/a n/a	9:1 0.96 [0.09, 10.45]
Family history of food allergy (yes:no) Odds ratio 95% CI	1:4 1.04 [0.10, 10.77]	2:6 1.5 [0.24, 9.34]
Family history of environmental allergy (yes:no) Odds ratio 95% CI	1:4 0.63 [0.6, 6.30]	4:4 3.57 [0.71, 18.04]
Family history of asthma (yes:no) Odds ratio 95% CI	2:4 3.1 [0.44, 21.63]	4:5 8.0 [1.36, 47.02]
Family history of eczema (yes:no) Odds ratio 95% CI	3:4 2.25 [0.42, 12.03]	5:5 3.71 [0.83, 16.55]

*CI* confidence interval

**Fig. 2:**
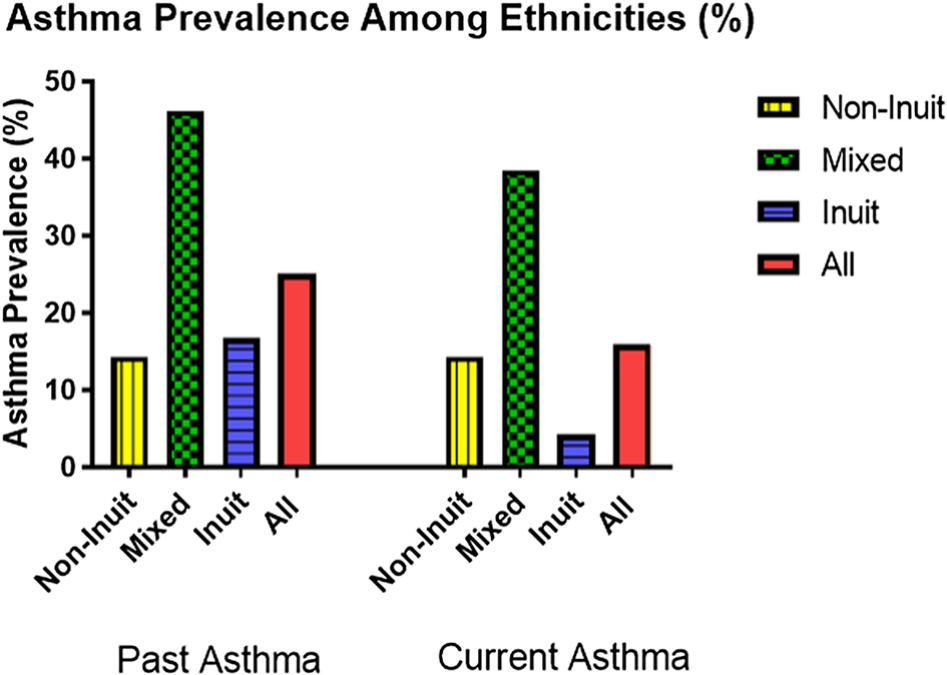
Asthma prevalence among Grade 1 children attending schools in Iqaluit

One quarter of the 44 participants had a previous history of asthma, highest among the mixed ethnicity at 46.2% and lowest among the non-Inuit at 14.3% Inuit at 16.7% (Fig. 2).

### Prevalence of allergic rhinitis

**Fig. 3:**
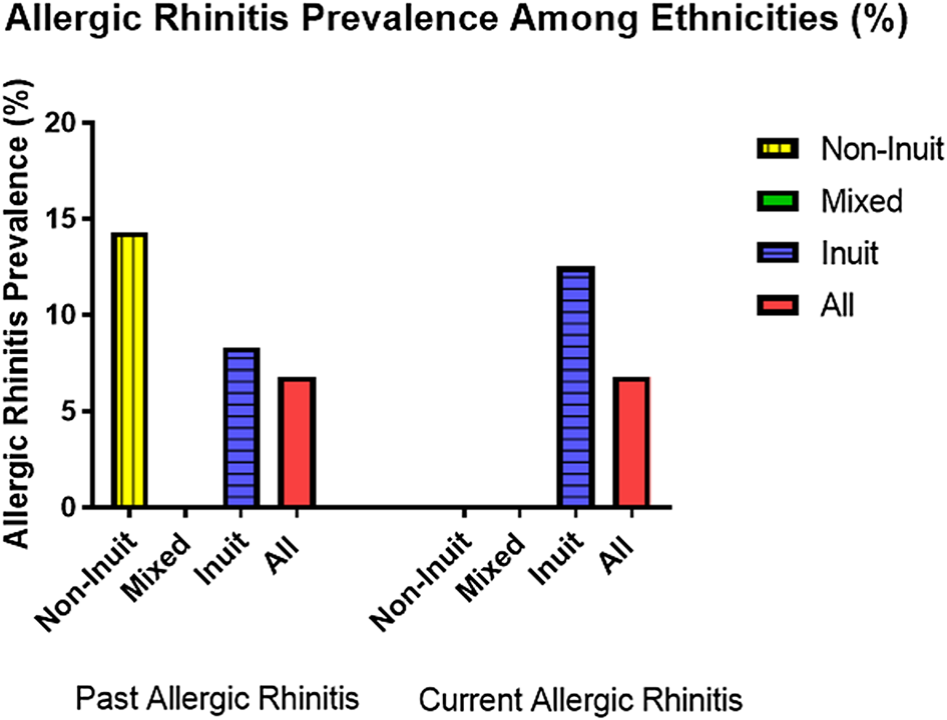
Allergic rhinitis prevalence among Grade 1 children attending schools in Iqaluit

The prevalence of both past and current allergic rhinitis was 6.8% (Fig. 3).

Interestingly, only Inuit children had a current diagnosis of allergic rhinitis, whereas both Inuit and non-Inuit children only had a past history consistent with allergic rhinitis. The prevalence of current allergic rhinitis amongst Inuit children was 12.5% of the enrolled Inuit students. The male to female ratio was 2:1. The overall prevalence of past allergic rhinitis was 6.8% with two-thirds of the cases amongst Inuit children and one-third amongst non-Inuit. Prevalence of past or current allergic rhinitis amongst mixed-ethnicity children was zero. The prevalence of past allergic rhinitis among the non-Inuit was higher than the Inuit, 14.3% compared to 8.3%. The male to female ratio was 2:1 (Table 2).

**Table 2:** Summary of the findings in relation to current and past allergic rhinitis

The questionnaire was completed by 44 subjects	Current allergic rhinitis	Past allergic rhinitis
Male:female Odds ratio 95% CI	2:1 1.42 [0.12, 16.91]	2:1 1.42 [0.12, 16.91]
Ethnicity		
Inuit, n (%) Mixed, n (%) Non-Inuit, n (%) Total, n (%) Chi square (p-value)	3 (12.5) 0 0 3 (6.8) 1.69 (0.56)	2 (8.3) 0 1 (14.3) 3 (6.8) 1.78 (0.41)
Smoker (yes:no) Odds ratio 95% CI	2:1 3.86 [0.32, 46.32]	1:2 0.87 [0.07, 10.38]
Crowdedness correlation (p)	− 0.225 (0.14)	− 0.176 (0.25)
Cat owner (yes:no) Odds ratio 95% CI	0:0 n/a n/a	1:0 n/a n/a
Dog owner (yes:no) Odds ratio 95% CI	2:0 n/a n/a	1:0 n/a n/a
Exclusive breast feeding (yes:no) Odds ratio 95% CI	1:2 0.29 [0.02, 3.46]	1:2 0.29 [0.02, 3.46]
Being ever outside Nunavut (yes:no) Odds ratio 95% CI	3:0 n/a n/a	3:0 n/a n/a
Previous respiratory hospitalization (yes:no) Odds ratio 95% CI	1:2 2.06 [0.17, 25.68]	1:2 2.06 [0.17, 25.68]
TB vaccination (yes:no) Odds ratio 95% CI	3:0 n/a n/a	3:0 n/a n/a
Family history of food allergy (yes:no) Odds ratio 95% CI	1:1 4.57 [0.25, 82.25]	0:2 n/a n/a
Family history of environmental allergy (yes:no) Odds ratio 95% CI	1:1 2.80 [0.16, 49.10]	0:2 n/a n/a
Family history of asthma (yes:no) Odds ratio 95% CI	2:1 13.6 [1.03, 179.03]	1:2 2.75 [0.21, 35.33]
Family history of eczema (yes:no) Odds ratio 95% CI	2:1 6.0 [0.49, 73.45]	1:2 1.32 [0.11, 16.04]

*CI* confidence interval

### Prevalence of eczema

**Fig. 4:**
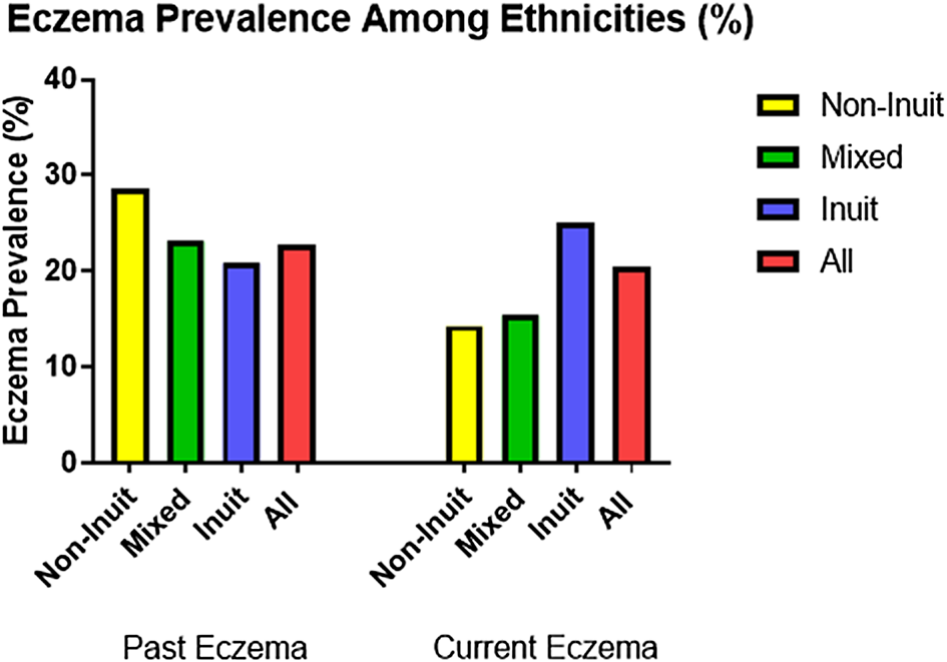
Eczema prevalence among Grade 1 children attending schools in Iqaluit

The prevalence of current eczema amongst the 44 Grade 1 children was 20.5%, with two-thirds of the cases amongst Inuit children (Fig. 4). Specifically, prevalence amongst Inuit, mixed ethnicity and non-Inuit was as follows 25, 15.4 and 14.3%. The male to female ratio was 1:1.25. In contrast, prevalence of past eczema amongst Inuit, mixed ethnicity and non-Inuit were as follows, 20.8, 23.1 and 28.5%, respectively. The male to female ratio was 1:1 (Table 3).

**Table 3:** Summary of the findings in relation to current and past eczema

The questionnaire was completed by 44 subjects	Current eczema	Past eczema
Male:female Odds ratio 95% CI	4:5 0.47 [0.11, 2.08]	1:1 0.62 [0.15, 2.56]
Ethnicity		
Inuit, n (%) Mixed, n (%) Non-Inuit, n (%) Total, n (%) Chi square (p-value)	6 (25) 2 (15.4) 1 (14.3) 9 (20.5) 0.58 (0.88)	5 (20.8) 3 (23.1) 2 (28.6) 10 (22.7) 0.45 (0.89)
Smoker (yes:no) Odds ratio 95% CI	3:6 0.85 [0.18, 3.97]	4:6 1.22 [0.29, 5.20]
Crowdedness correlation (p)	− 0.135 (0.38)	0.047 (0.76)
Cat owner (yes:no) Odds ratio 95% CI	0:2 n/a n/a	1:3 3.0 [0.20, 44.36]
Dog owner (yes:no) Odds ratio 95% CI	6:2 7.13 [1.18, 43.14]	5:3 3.33 [0.65, 17.18]
Exclusive breast feeding (yes:no) Odds ratio 95% CI	4:5 0.42 [0.09, 1.85]	6:4 0.93 [0.22, 3.93]
Being ever outside Nunavut (yes:no) Odds ratio 95% CI	7:2 1.26 [.22, 7.23]	9:1 3.91 [.44, 35.15]
Previous respiratory hospitalization (yes:no) Odds ratio 95% CI	3:6 2.42 [0.47, 12.47]	3:7 2.00 [0.40, 10.05]
TB vaccination (yes:no) Odds ratio 95% CI	8:0 n/a n/a	9:1 0.96 [0.09, 10.45]
Family history of food allergy (yes:no) Odds ratio 95% CI	2:5 1.87 [0.29, 12.01]	2:6 1.5 [0.24, 9.34]
Family history of environmental allergy (yes:no) Odds ratio 95% CI	3:4 2.34 [0.43, 12.77]	5:3 7.22 [1.34, 38.92]
Family history of asthma (yes:no) Odds ratio 95% CI	3:5 4.5 [0.77, 26.45]	3:6 3.63 [0.64, 20.57]
Family history of eczema (yes:no) Odds ratio 95% CI	5:4 4.82 [1.02, 22.84]	6:4 6.75 [1.44, 36.60]

*CI* confidence interval

### Prevalence of reported food and environmental allergy

**Fig. 5:**
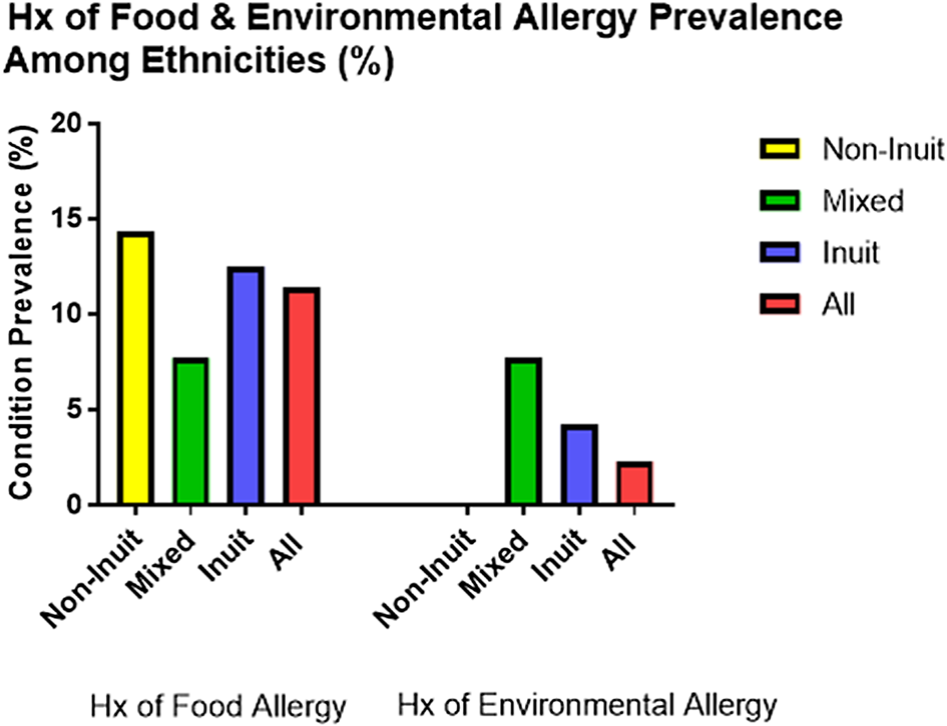
History of food and environmental allergies among Grade 1 children attending schools in Iqaluit

The prevalence of a history of food allergy as reported by parents was 11.4%, with the highest prevalence among the non-Inuit at 14.3% followed by the Inuit at 8.3% (Fig. 5). Whereas, prevalence of food allergy amongst the mixed population was 7.7%. The male to female ratio was 1:1.5. On the contrary, the prevalence of a history of environmental allergy as reported by parents was 4.5%, none of them were non-Inuit children, while the prevalence among the mixed ethnicity was 7.7% and among the Inuit 4.2%. The male to female ratio was 1:1 (Table 4).

**Table 4:** Summary of the findings in relation to reported history of food and environmental allergies

The questionnaire was completed by 44 subjects	Reported food allergy	Reported environmental allergy
Male:female Odds ratio 95% CI	2:3 0.42 [0.06, 2.79]	1:1 0.68 [0.04, 11.63]
Ethnicity		
Inuit, n (%) Mixed, n (%) Non-Inuit, n (%) Total, n (%) Chi square (p-value)	3 (12.5) 1 (7.7) 1 (14.3) 5 (11.4) 0.559 (1.0)	1 (4.2) 1 (7.7) Zero 2 (4.5) 0.913 (1.0)
Smoker(s) at home (yes:no) Odds ratio 95% CI	3:2 3.00 [0.45, 20.24]	1:1 1.80 [0.11, 30.90]
Crowdedness correlation (p)	− 0.172 (0.27)	− 0.128 (0.41)
Cat ownership (yes:no) Odds ratio 95% CI	1:2 4.75 [0.29, 78.74]	0:0 n/a n/a
Dog ownership (yes:no) Odds ratio 95% CI	1:2 0.73 [0.06, 8.92]	1:0 n/a n/a
Exclusive breast feeding (yes:no) Odds ratio 95% CI	2:3 0.37 [0.06, 2.51]	0:2 n/a n/a
Being ever outside Nunavut (yes:no) Odds ratio 95% CI	4:1 1.43 [0.14, 14.35]	2:0 n/a n/a
Previous respiratory hospitalization (yes:no) Odds ratio 95% CI	1:4 0.97 [0.10, 9.91]	2:0 n/a n/a
TB vaccination (yes:no) Odds ratio 95% CI	5:0 n/a n/a	2:0 n/a n/a
Family history of food allergy (yes:no) Odds ratio 95% CI	0:4 n/a n/a	0:0 n/a n/a
Family history of environmental allergy (yes:no) Odds ratio 95% CI	0:4 n/a n/a	0:0 n/a n/a
Family history of asthma (yes:no) Odds ratio 95% CI	2:3 4.27 [0.57, 32.24]	1:0 n/a n/a
Family history of eczema (yes:no) Odds ratio 95% CI	1:4 0.61 [0.06, 6.13]	2:0 n/a n/a

*CI* confidence interval

### Prevalence of smoking and hospitalization due to a respiratory condition

**Fig. 6:**
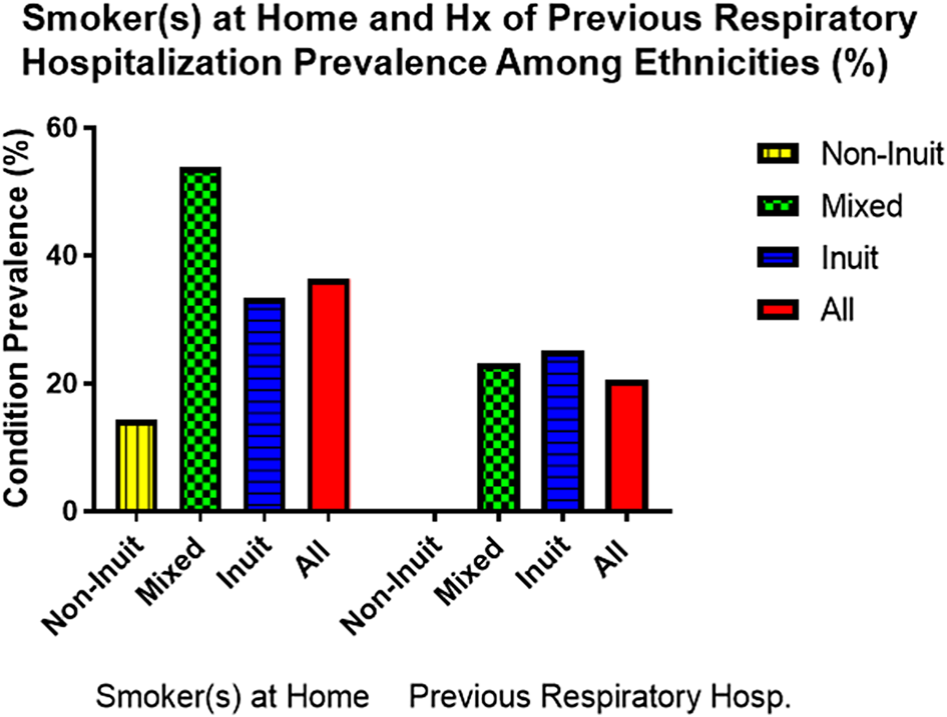
The prevalence of having smoker(s) at home and history of previous respiratory hospitalization among Grade 1 children attending schools in Iqaluit

Having at least one smoker living in the household was 36.4% with the highest rate among the mixed ethnicity at 53.8% while lower among the Inuit at 33.3% and the least among non-Inuit at 14.3% (Fig. 6). No statistically significant relationship was found between current or past asthma and having a smoker at home.

None of the non-Inuit students have been previously hospitalized due to bronchiolitis or respiratory infections, on the other side, one-quarter of the Inuit students had a history of at least one admission and a similar rate of 23.1% among the mixed ethnicity.

### Prevalence of breast feeding

Almost two-thirds (61.4%) of the children were exclusively breastfed during the first 4 months of life, with a higher percentage among the non-Inuit children at 71.4% while 61.5% among the mixed ethnicity and a little bit lower among the Inuit students at 58.3%. No statistically significant relationship was found between exclusive breastfeeding during the first 4 months of life and asthma, allergic rhinitis or eczema.

### Pet ownership

**Fig. 7:**
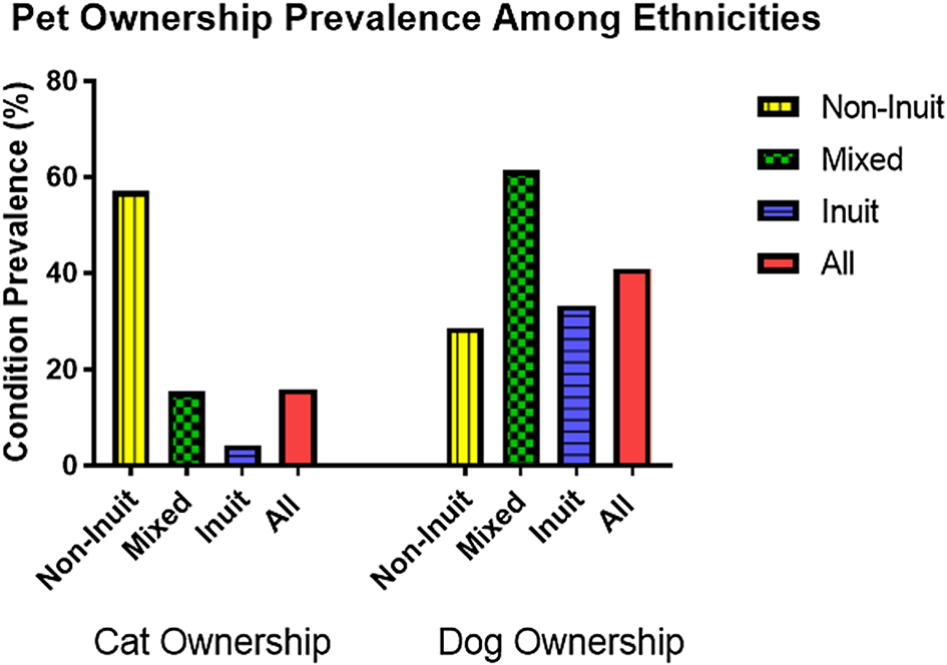
The prevalence of having a pet among Grade 1 children attending schools in Iqaluit

Having a dog at home was relatively common at 40.9% with the highest rate among the mixed ethnicity at 61.5% followed by the Inuit at 33.3% and lastly by the non-Inuit at 28.6% (Fig. 7). A completely different picture was noticed in regard to having a cat at home with a general rate of 15.9%, with the highest among the non-Inuit at 57% compared with the Inuit at 4.2% while the mixed ethnicity at 15.4%.

No statistically significant relationship was found between pet ownership and asthma or allergic rhinitis. Three-quarters of those with current eczema had a dog at their home (OR 7.13, CI 1.18–43.14). Interestingly, none of those with current eczema have a cat at home. Almost two-thirds (62%) of those with past eczema had a dog at their home while only a quarter have a cat.

Having a family history of eczema was a risk factor to had a current eczema (OR 4.82, CI 1.02–22.84) and past eczema (OR 6.75, CI 1.44–31.60).

Family history of environmental allergy was associated with having past eczema (OR 4.39, CI 1.26–15.37).

No statistically significant relationship was found between exclusive breastfeeding during the first 4 months of life and having current or past eczema.

None of those who were not BCG vaccinated had current eczema. (OR 0.78, CI 0.66–0.93).

### Prevalence of sensitization to the tested food

The prevalence of allergy to egg white as confirmed by history and skin prick testing is 3.3%, with that single case being a non-Inuit. The prevalence of allergy to peanut as confirmed by history and skin prick testing is 3.3%, with that single case being an Inuit. The prevalence of allergy to tree nuts as confirmed by history and skin prick testing is 6.7%, none of them was non-Inuit. None of those that were allergic to egg white, peanut or tree nut had current asthma or eczema. No child was sensitized to cow’s milk, soy or wheat.

### Prevalence of sensitization to the tested environmental allergens

The prevalence of sensitization to cat was surprisingly high at 26.7% with the highest prevalence among the non-Inuit at 50% followed by the Inuit at 23.5% then the mixed ethnicity at 14.3%. The prevalence of sensitization to tree pollen was 13.3%, none of them was non-Inuit with the highest prevalence among the mixed ethnicity at 42.9% while only 5.9% among the Inuit (Fisher’s exact test 5.130, p = 0.06). On the other hand, the prevalence of sensitization to grass was low at 6.7%, with higher prevalence among the mixed ethnicity at 14.3% compared to 5.9% among the Inuit (Table 5). No child was sensitized to house dust mite, mold, weed, ragweed or dog.

**Table 5:** Summary of the findings in relation to positive skin prick tests (environmental)

	Skin prick test tree	Skin prick test grass	Skin prick test cat
Male:female Odds ratio 95% CI	3:1 2.20 [0.20, 24.09]	2:0 n/a n/a	5:3 1.15 [0.22, 6.10]
Ethnicity			
Inuit, n (%) Mixed, n (%) Non-Inuit, n (%) Total, n (%) Chi square (p-value)	1 (5.9) 3 (42.9) 0 4 (13.3) 5.13 (0.06)	1 (5.9) 1 (14.3) 0 2 (6.7) 1.30 (0.69)	4 (23.5) 1 (14.3) 3 (50.0) 8 (26.7) 2.15 (0.39)
Smoker(s) at home (yes:no) Odds ratio 95% CI	1:1 1.6 [0.19, 13.24]	1:1 1.5 [0.09,27.36]	3:5 0.87 [0.16, 4.58]
Crowdedness correlation (p)	0.010 (0.95)	− 0.160 (0.40)	0.010 (0.94)
Cat ownership (yes:no) Odds ratio 95% CI	0:0 n/a n/a	0:1 n/a n/a	2:1 24.0 [1.03, 560.2]
Dog ownership (yes:no) Odds ratio 95% CI	3:0 n/a n/a	1:1 1.20 [0.07, 21.72]	5:1 10.00 [0.94, 105.92]
Exclusive breast feeding (yes:no) Odds ratio 95% CI	1:3 0.29 [0.03, 3.12]	0:2 n/a n/a	1:1 1.0 [0.20, 5.05]
Being ever outside Nunavut (yes:no) Odds ratio 95% CI	3:1 1.33 [0.12, 14.87]	0:2 n/a n/a	7:1 4.0 [0.41, 38.65]
Previous respiratory hospitalization (yes:no) Odds ratio 95% CI	1:1 7.67 [0.77, 76.45]	0:2 n/a n/a	1:7 0.64 [0.06, 6.80]
TB vaccination (yes:no) Odds ratio 95% CI	3:1 0.14 [0.01, 2.80]	2:0 n/a n/a	7:0 n/a n/a
Family history of food allergy (yes:no) Odds ratio 95% CI	1:1 4.20 [0.22, 79.32]	0:2 n/a n/a	1:6 0.53 [0.05, 5.55]
Family history of environmental allergy (yes:no) Odds ratio 95% CI	1:1 3.17 [0.17, 58.70]	0:2 n/a n/a	0:7 n/a n/a
Family history of asthma (yes:no) Odds ratio 95% CI	1:2 2.10 [0.16, 28.02]	0:2 n/a n/a	0:7 n/a n/a
Family history of eczema (yes:no) Odds ratio 95% CI	1:1 3.17 [0.17, 58.70]	0:2 n/a n/a	0:7 n/a n/a

*n/a* not applicable, *CI* confidence interval

### Environmental exposures

Almost three-quarters of the 44 Grade 1 children (72.7%) had been at least once outside Nunavut. The rate was double amongst male students compared to females, with the highest, as expected, among the non-Inuit children at 100% with mixed ethnicity at 92.3% and Inuit lowest at 54%.

Those children with current asthma were less likely to have never been outside Nunavut [11 out of 43 were never outside Nunavut and has no current asthma, OR 0.78, CI 0.65–0.94; and the same applies to those with past asthma (OR 0.66, CI 0.51–0.84)] (Table 1).

Three-quarters of those sensitized to trees were ever outside Nunavut while none of those sensitized to grass were ever outside Nunavut. 88% of those sensitized to cat have were outside Nunavut.

The average figure for crowdedness index at households (the number of tenants divided by the number of bedrooms) was about 1.65 for all the students enrolled in the study, with both the non-Inuit and mixed ethnicities below that average at 1.37 and 1.49 respectively while the Inuit students lived in a more crowded houses with the average index of 1.82. The crowdedness tended to be inversely related to having past asthma (Pearson correlation 0.285, p = 0.061) (Table 1).

Ninety percent of the students were vaccinated against TB, this included 100% of the Inuit children, 84.6% of the mixed ethnicity and 71.4% of the non-Inuit students. Having the BCG vaccine was associated with having current asthma, none of those who were not BCG vaccinated (4 students) had current asthma (OR 0.84, CI 0.73–0.97).

No statistically significant relationship was found between family history of asthma and having current asthma, while there was a positive relationship between having a family history of asthma and having past asthma (OR 8.0, CI 1.36–47.02).

Interestingly, none of those reported to have food allergy had a family history of food allergy (OR 1.18, CI 1.02–1.36).

No statistically significant relationship was found between exclusive breastfeeding during the first 4 months of life and the various studied allergy conditions. Though, it is worth mentioning that three-quarters of those sensitized to trees and all those sensitized to grass, peanut and tree nuts were not exclusively breastfed during the first 4 months of life.

All those sensitized to tree pollen have a dog and 83% of those sensitized to cat have a dog at home. Only a quarter of those sensitized to cat have a cat at home while two-thirds of those having a cat at home are sensitized to cats (OR 24, CI 1.03–560.18).

One-third of those sensitized to trees had a family history of asthma while none of those sensitized to grass had a family history of asthma. None of those sensitized to cat had a family history of asthma (OR 1.44, CI 1.10–1.88).

Sensitization to trees was highly associated with having current asthma, eczema, and allergic rhinitis, while sensitization to grass was only associated with having current eczema. The sensitization to cats was associated more with current eczema followed by asthma and lastly allergic rhinitis.

All those found to be allergic to egg white, peanut and tree nut had no family history of food or environmental allergy. Similarly, none of those sensitized to grass had a family history of environmental allergy and none of those sensitized to cat had a family history of environmental allergy (OR 1.54, CI 1.12–2.12).

## Discussion

For the first time, our study shows that the prevalence of asthma and allergic rhinitis among children, aged 6–7 years, in Iqaluit is 15.9 and 6.8%, respectively. Interestingly, this is not substantively different from the prevalence of these conditions in the rest of Canada (18.2 and 10.8%, respectively) [24]. The ethnic distribution of the participants in this study was representative of the actual ethnic distribution in the city of Iqaluit. Of those who completed the questionnaire 54.5% were Inuit, 29.5% were of mixed ethnicity and 15.9% were non-Inuit. Statistics Canada [25] considers any individual who has at least one parent with Inuit ancestry as an Inuit. If the above classification was adopted in our study this would suggest that 84% among the participants were Inuit, which goes with their percentage in the city of Iqaluit [2]. The findings in this study do show a much higher prevalence of asthma and special characteristics that define this mixed ethnicity group in relation to lifestyle (crowdedness, passive smoking, ever being outside Nunavut and dog ownership). However, it must be noted that the non-Inuit ethnicity does not actually represent a single ethnicity, it is mainly comprised of White Canadians (most originate from western Europe) in addition to a few other ethnicities (personal observation).

In this study, the prevalence of current asthma was statistically significantly different among the three ethnic groups, Inuit, mixed ethnicity and non-Inuit with the highest among the mixed ethnicity group at 38.5% while the least among the Inuit group at 4.2%. Asthma was reported at a similar low rate of 4% among the Inuit children as documented by the 2007–2008 health survey which also relied on parental reporting [26]. Furthermore, Chang et al. found that children with Inuit ancestry living outside Nunavut had a significantly lower prevalence of asthma than those with North American Indian and Métis ancestries [16]. The prevalence of current and ever asthma among all Aboriginal children combined was 5.7 and 14.3%, respectively. Also, similar to our study findings, the prevalence of ever asthma was greater in boys than in girls [16]. The findings of this study are in agreement with previously reported studies, where Aboriginal children (5.7%) had significantly lower levels of asthma prevalence than non-Aboriginal children (10.0%) in northern Canada [27].

The reported prevalence of lifetime wheeze among children in the southern warmer Canadian city of Toronto was 29.2%, while 14.2% reported wheeze in the past 12 months [28]. Habbik et al. has shown wide variation in asthma prevalence among Grade 1 children in different Canadian cities. Lifetime prevalence of asthma was documented as 17.2% in Hamilton and 11.2% in Saskatoon. The prevalence of wheezing in the 12 months before the survey among the children aged 6–7 years was 20.1% in Hamilton and 14.1% in Saskatoon, with the male to female ratio of 1.5:1 [29].

Interestingly, self-reported asthma among school children living in another arctic region, Norway, was similar to the prevalence observed among the non-Inuit population in Nunavut in our study [8]. A similar (to the Inuit) low rate of asthma (3.9%) was reported in Nikel, a Russian arctic city [30].

The prevalence of current allergic rhinitis is low at 6.8%, all of the reported cases were Inuit and constituting 12.5% of the enrolled Inuit students with male predominance, which is less than the reported 14.6–22.6% found in five other southern Canadian cities [12]. This difference is most likely due to less allergen exposure in Nunavut and that the above study was conducted among older children (13–14 year-old). Again, our findings were similar to the low rate of allergic rhinitis (13.9%) that was reported in Nikel, a Russian arctic city [30]. This disparity in regional variations in the prevalence rates suggests dissimilar risk factors for the development or expression of wheezing (asthma), allergic rhinitis and atopic eczema [12].

The prevalence of past allergic rhinitis was also 6.8% but being higher among the non-Inuit compared to the Inuit, 14.3 and 8.3% respectively with male predominance as well. This higher prevalence of previous allergic rhinitis among the non-Inuit could be attributed to transient symptoms when travelling outside Nunavut or being living outside Nunavut earlier in life because of an exposure to a triggering allergen that is absent or less abundant in Nunavut. It may also be due to a different mechanism behind the development of those symptoms, knowing that the non-Inuit showed less sensitization to most of the major environmental allergens that exist in the south.

Repeated cross-sectional surveys in a subarctic population in northern Norway between 1985 and 2008 demonstrated an increase in the prevalence of asthma and allergic rhinoconjunctivitis ever among school children (7–14 years) [11], this emphasizes the need for follow-up studies.

Our study findings go with the Kovesi et al. finding that the Inuit infants have extremely high rates of lower respiratory tract infections with 25% of the children had, at some time, been hospitalized for chest illness [31], interestingly, this was not associated with higher prevalence of subsequent asthma.

**Fig. 8:**
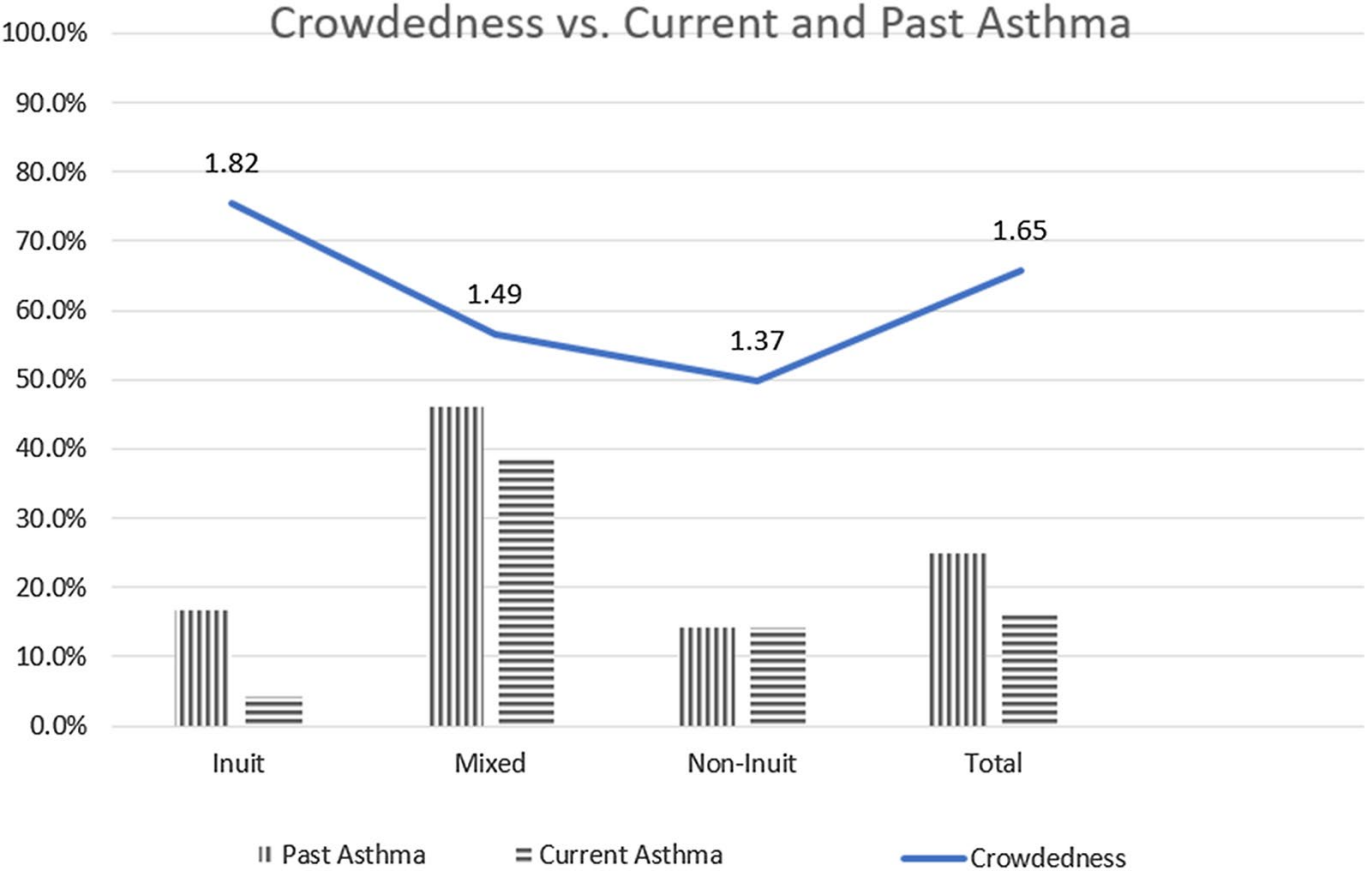
Crowdedness versus current and past asthma

The finding that the Inuit are living in more crowded houses was not a surprise, a previous study of the indoor quality of Inuit houses in Nunavut have found that the mean number of occupants per house in Nunavut was 6.1 people [32]. The higher crowdedness being associated with not having previous asthma, see Fig. 8. While the hygiene hypothesis can be used to explain this; having a crowded household increases the respiratory infections and decreases asthma, on the other hand, such a higher rate of respiratory infections is expected to lead to more wheezes earlier in life which may lead to either a higher prevalence of atopy if infections were mainly RSV and HRV (especially HRV C as suggested in the COAST study) [33] or may have led to lower rates if the crowding in the home created an environment of increased endotoxin/LPS with extensive dog exposure as in the Bavarian farm families with their bovine exposure [34].

All the non-Inuit children and the vast majority of the mixed ethnicity students were ever outside Nunavut, compared to half of the Inuit. This might have a role in the degree of exposure to a higher concentration of environmental allergens in the South which might explain the significant relation between having current asthma, past asthma and being ever outside Nunavut (OR 0.78, CI 0.65–0.94) and (OR 0.66, CI 0.51–0.84) respectively. The questionnaire did not explore the timing, duration, purpose or frequency of being ever outside Nunavut. So there is not enough information to determine a causality relationship versus being a confounding factor.

A possible explanation for the relationship between the family history of asthma and past asthma but not with the current asthma is having a sibling with recurrent wheezes related to respiratory illnesses or having passive smoking with the development of wheezes among siblings, so it might be a confounding factor rather than a genetic predisposition.

The current eczema prevalence amongst the three ethnic groups was 20.5%, with the highest prevalence recorded among the Inuit at 25% compared to 15.4% among the mixed ethnicity. While 14.3% of non-Inuit recorded a current history of eczema, with more cases among the females. Interestingly, the Mushuau Inuit of Natuashish (a minority of Inuit people living in Labrador, Canada) recorded a prevalence of 16.5%, with female predominance as well (female to male ratio 2:1) [13]. In agreement with our study findings, Weiland et al. have previously reported that the prevalence of eczema symptoms correlated positively with latitude and negatively with the mean annual outdoor temperature [10], thus, making it an expected finding to get a higher eczema rate in Nunavut compared to that in southern Canada. Similarly, a high prevalence of eczema (23.6%) was reported in Arctic Norway [8] compared to a lower prevalence of 12.7% in the southern part of the same country [35, 36]. Also, in Sweden, the prevalence of atopic eczema among 7–8-year old children was reported to be higher in Kiruna in the North than in Gothenburg in the South (23% vs 18.7%) with a similar higher female to male ratio [37].

The higher prevalence of past eczema among the non-Inuit compared to the current one being highest among the Inuit might indicate that eczema is more transient among the non-Inuit while persists for a longer time in Inuit children. According to unpublished clinical experience in Nunavut, the Inuit children tend to have more severe eczema, whether this is related to genetic predisposition, access to medical care, treatment compliance, household conditions or a combination of these factors is worth to be explored. That was out of the scope of this current study. The estimates of the annual costs of eczema in Canada is about $1.4 billion [38] which imposes a considerable financial burden on Canadian society especially among the lower socioeconomic populations in the far North.

Having a dog at home in Iqaluit is relatively common, especially among the mixed ethnicity while having a cat was the highest among the non-Inuit. There are likely a number of reasons for this; but for centuries, dogs have been used by the Inuit for work and transportation. While cats are generally not perceived to be of substantive value to the inhabitants in the Nunavut environment. In Nunavut dogs tend to be outside of the home. Owning a dog was strongly associated with the presence of current eczema (OR 7.13, CI 1.18–43.14) There was no relationship with cat ownership. These data are contrary to previous reports indicating that dog ownership significantly reduced the risk of eczema at the age of 4 years among dog-sensitized children while cat ownership combined with cat sensitization significantly increased the risk [39], though in that same article it was mentioned that children exposed to the highest dog allergen concentrations had a significantly lower risk of eczema. This is not the case in our study population, since most of the dogs are held outside the houses in Nunavut while the cats are indoors most of the time.

As expected, the study found a high correlation between having a family history of eczema and current eczema as well as past eczema. Whether this is related to atopy inheritance, household environment, exposure to the same risk factors or a familial skin barrier issue like the filaggrin mutation could not be determined in this study.

No significant relationship was found between eczema and having a family history of food allergy, which is reasonable taking into consideration the various mechanisms (intrinsic vs extrinsic) of developing those atopic diseases, especially when studied among different ethnicities.

A significant relationship was found between family history of environmental allergies and having past eczema but not with the current eczema, an explanation could be related to the fact that the current eczema was profoundly prominent among the Inuit children with less expected family history of environmental allergies because of low allergen exposure while the past eczema was more among the non-Inuit students whom their parents most probably were born and raised outside Nunavut with more allergen exposure. The study questionnaire did not address neither the place of birth of the child nor the parents.

A previous study by McIsaac [40] found that only 24.8% of the Inuit were exclusively breastfed to 6 months which is much lower than our finding, possibly because our study asked only about the first 4 months of life. There have been many studies with conflicting results in regard to a possible atopy protective effect of exclusive breastfeeding for the first 4–6 months, neither the study design nor the sample size can allow taking a side on this continued debate.

Our study shows a surprisingly high prevalence of sensitization among children, aged 6–7 years, in Iqaluit to cats, at 26.7%, which is much higher that the 8.6% prevalence among Grade 1–8 children in the province of Saskatchewan [41].

Equally surprising was the prevalence of sensitization with suggestive history of allergy to peanut and tree nut among children aged 6–7 years in Iqaluit at 3.3 and 6.7%. Especially given a prevalence of peanut allergy as tested in Montreal among children aged 4–9 years was 1.62% [42]. However, this is a small sample and food challenges were not performed.

All the non-Inuit children and the vast majority of the mixed ethnicity students were ever outside Nunavut, compared to half of the Inuit. This might have a role in the degree of exposure to a higher concentration of environmental allergens in the South which might explain the significant relation between having current asthma, past asthma and being ever outside Nunavut (OR 0.78, CI 0.65–0.94) and (OR 0.66, CI 0.51–0.84) respectively. The questionnaire did not explore the timing, duration, purpose or frequency of being ever outside Nunavut. Many of the Inuit children get outside Nunavut for the first time because of being urgently transferred to a tertiary hospital in Ontario or Manitoba (no tertiary hospital or pediatric intensive care unit in all the territory), one of the common conditions for such an urgent transfer is having a severe lower respiratory tract infection (personal observation as a pediatrician locum). So there is no enough information to determine a causality relationship versus being a confounding factor.

The prevalence of sensitization to grass being highest among the mixed ethnicity might have a similar explanation though it is worth mentioning that two of the grasses used in the skin test [June (*P. pratensis*) and Redtop (*A. alba*)] can be found in Nunavut [43, 44] which might explain the sensitization among children that have never been outside Nunavut, when compared to Greenland Inuit, the Inuit children in Greenland were more likely to be sensitized to inhaled allergens compared to the non-Inuit, with grass being the major inhaled allergen [44].

The absence of sensitization to house dust mite, mold, weed, ragweed and dog in our study is consistent with the findings in Greenland where the prevalence of sensitization to house dust mite is four times lower than that in Denmark, probably because of lower indoors house dust mite count similar to northern Norway and Iceland [45]. The overall prevalence of at least one positive skin prick test was 22.8% in Denmark compared to only 6.4% in Uummannaq (a northern community in Greenland with similarities to Iqaluit). In Denmark, the total birch pollen counts were 40–1000 times higher compared to Nuuk (Greenland), whereas the grass pollen count was 13–30 times higher in Denmark compared to Nuuk. In Denmark, house dust mites were found in 72% of households (> 10/0.1 g dust) while less than 15% of households in Greenland had measurable levels of house dust mites. Similar to my personal observation in Iqaluit, that study reported that dogs were held indoor much less frequently in Uummannaq [46], making the expected dog allergen load low inside the houses.

A study in northern Norway had similar findings with low house dust mite and mold sensitization [47], another one in northern Sweden showed that sensitization to cats was the most common at 19% while sensitization to mites and mold was uncommon [48].

A previous house survey in Nunavut have shown that although building’s fungal concentrations were low, the mattress fungal levels were markedly increased, and the dust mites were virtually non-existent [31].

The strength of this study includes using a validated ISAAC questionnaire with the added relevant questions to this population and geographical area.

Limitations of this study were related mainly to the small sample size which constituted one-third of the targeted cohort. This may relate to suspicion among the families about clinical research environment which has not been conducted in this part of Canada for decades. Hopefully, this study will be a breakthrough in regard to building a positive relationship and trust with the local community in Nunavut and encourage further acceptance of research.

Given the small sample size, a post hoc power analysis revealed low power in relation to most associations, we acknowledge this limitation (Table 6, Additional file 4: Appendix S4). There is a possibility that atopy-positive families were more willing to participate. Thus, selection bias could have been possible. As well, recall bias remains a possibility as with every retrospective questionnaire-based study. Also, the study has its limits in regard to determining associations but not causality relationships. A larger scale longitudinal study is highly recommended to avoid such limitations.

Despite those limitations, we think that our study contributes to the existing literature because of the scarcity of data about allergies and allergic conditions among the Inuit population and other ethnicities living in this part of the World. Add to this, such unique baseline data serve as a benchmark for future prevalence studies.

## Conclusion

Variations in the prevalence and risk factors of asthma, allergic rhinitis, eczema and sensitization among different ethnicities living at the same subarctic environment may be related to genetic, gene-environment interaction and/or lifestyle factors that require further investigation.

## Additional files

**Additional file 1: Appendix S1.** Study Questionnaire.

**Additional file 2: Appendix S2.** Added questions to the ISAAC Questionnaire.

**Additional file 3: Appendix S3.** Tested allergens.

**Additional file 4: Appendix S4.** Post hoc power analysis findings.

## Abbreviations

ISAAC: International Study of Allergy and Asthma in Children
SPT: skin prick test
sq. km: square kilometer
TB: tuberculosis
BCG: Bacille Calmette–Guérin
HDM: house dust mite
OR: odds ratio
CI-95: confidence interval of 95%
AR: allergic rhinitis
L/s: liter per second
RSV: respiratory syncytial virus
HRV: human rhinovirus
LPS: lipopolysaccharide
