# Supplementary material for: Evaluation of eczema, asthma, allergic rhinitis and allergies among the Grade-1 children of Iqaluit
Source: Allergy Asthma Clin Immunol. 2018 Feb 27;14:9. doi: 10.1186/s13223-018-0232-2 (PMC5827980; doi:10.1186/s13223-018-0232-2)
Supplement: Supplementary file 1 — Additional file 1: Appendix S1. Study Questionnaire. [file 13223_2018_232_MOESM1_ESM.docx]

Appendix-1.

“Study Questionnaire” (version 2.0, September 2015)

**Evaluation of Eczema, Asthma and Allergies among the Children of Iqaluit (EAACI) Study**

**Study Questionnaire**

Child’s name:______________________ Date of birth:__________________

Gender:___________ *Day/month/year*

Name of the parent/guardian filling the questionnaire______________________

Relationship to the child________________________

**1- Is one of the biological parents or both Inuk? Please check one answer**:

( ) Yes, both biological parents are Inuk

( ) Yes, only one biological parent is Inuk

( ) No, both biological parents are NOT Inuk

**2- Does your child have allergies?**

( ) Yes, to____________________________________________________

( ) No

**3- If your child has an allergy, was he/she been tested for that?**

( ) Yes ( ) No

**4- Did your child ever have anaphylaxis (severe allergic reaction) or need to use EpiPen?**

( ) Yes, to ____________________, at what age? ___________

( ) No

**5- Did your child need any puffers during the last 12 months?**

( ) Yes ( ) No

**6- Did your child ever have whistling/wheezing of the chest at any time in the past?**

( ) Yes ( ) No

**7- Did your child ever have whistling/wheezing of the chest in the past 12 months?**

( ) Yes ( ) No

**8- In the past 12 months, how often has your child had his/her sleep been disturbed?**

( ) Occasional ( ) One night per week ( ) Two nights per week ( ) Three nights per week ( ) More than three nights per week.

**9- In the past 12 months, has your child’s wheezing ever been severe enough to limit speech?**

( ) Yes ( ) No

**10- In the past 12 months, has your child’s chest sounded wheezy during or after exercise?**

( ) Yes ( ) No

**11- In the past 12 months, did your child have a cough at night not associated with respiratory tract infection?**

( ) Occasional ( ) One night per week ( ) Two nights per week ( ) Three nights per week ( ) More than three nights per week.

**12- Did your child ever have a problem with sneezing/runny/blocked nose not associated with cold/flu?**

( ) Yes ( ) No

**13- In the past 12 months, did your child have nasal problems not associated with cold/flu?**

( ) Yes ( ) No

**14- In the past 12 months, was this nasal problem associated with itchy/watery eyes?**

( ) Yes ( ) No

**15- Did your child ever have hay fever (also known as allergic rhinitis)?**

( ) Yes ( ) No

**16- Did your child ever have an itchy, intermittent skin rash for at least 6 months?**

( ) Yes ( ) No

**17- Did your child have this rash in the past 12 months?**

( ) Yes ( ) No

**18- Did your child ever have Eczema (skin red itchy spots with dryness)?**

( ) Yes ( ) No

**19- If your child has eczema, is he/she currently using a steroid cream (like Hydrocortisone, Desonide, Betaderm,…)?**

( ) Yes

( ) No, but uses moisturizers (like Aveeno, Glaxal base, Cetaphil, Vaselin,…)

( ) No, does not use any cream.

**20- Did your child ever visit or live outside Nunavut?**

( ) Yes ( ) No

**21- Does your child eat the following types of foods or has eaten them in the past without an allergic reaction?**

Milk (regular or formula): ( ) Yes ( ) No

Eggs: ( ) Yes ( ) No

Peanut: ( ) Yes ( ) No

Tree nut (like Cashew, Pistachio, Pecan, Brazil nut): ( ) Yes ( ) No

Fish: ( ) Yes ( ) No

Shellfish (like Shrimp, Crab, Lobster): ( ) Yes ( ) No

**22- Do you have pets?**

A dog: ( ) Yes ( ) No

A cat: ( ) Yes ( ) No

Other pets ( ) Yes; Name it ___________ ( ) No

**23- Any smokers live in the same house? (even if they smoke only outside the house)** ( ) Yes ( ) No

**24- Family history: (Asking about biological parents/ brothers/ sisters) if the answer is Yes, please write next to it the relationship to the child like father, mother, brother or sister. *In case of an adopted/fostered child and you do not know the answer about the biological family please chose “I do not know”.***

Eczema ( ) Yes, ________________________ ( ) No ( ) I do not know

Asthma ( ) Yes, ________________________ ( ) No ( ) I do not know

Food Allergy ( ) Yes, ________________________ ( ) No ( ) I do not know

Environmental allergy like to cats, dogs, trees, house dust mite, mold, etc:

( ) Yes, to _____________________ ( ) No ( ) I do not know

**25- Was your child exclusively breast fed during the first 4 months of life?**

( ) Yes ( ) No ( ) I do not know

**26- Was your child given the TB vaccination at birth (also called BCG) to prevent a disease called Tuberculosis?**

( ) Yes ( ) No ( ) I do not know

**27- Was your child ever been hospitalized because of a lung infection or bronchiolitis?**

( ) Yes, how many times?__________________ ( ) No

**28- Number of people (adults and children including this child) living in the house:_________.**

**29- Number of bed rooms:___________.**

**30-The parent’s phone number in case we need to contact you:_____________________.**
