# Supplementary material for: Evaluation of eczema, asthma, allergic rhinitis and allergies among the Grade-1 children of Iqaluit
Source: Allergy Asthma Clin Immunol. 2018 Feb 27;14:9. doi: 10.1186/s13223-018-0232-2 (PMC5827980; doi:10.1186/s13223-018-0232-2)
Supplement: Supplementary file 2 — Additional file 2: Appendix S2. Added questions to the ISAAC Questionnaire. [file 13223_2018_232_MOESM2_ESM.docx]

Appendix-2 (The questions added to the ISAAC Study Questionnaire)

| **The added questions to ISAAC Study Questionnaire** | **Rationale to add** |
| --- | --- |
| Is one of the biological parents or both Inuk? | Find differences among different ethnicities living in the same subarctic environment |
| Does your child have allergies? | Know the current allergy prevalence based on parent’s perception |
| If your child has an allergy, was he/she been tested for that? | Try to get an idea about the possible actual prevalence of allergy |
| Did your child ever have anaphylaxis (severe allergic reaction) or need to use EpiPen? | To get an idea about the prevalence of anaphylaxis |
| Did your child ever visit or live outside Nunavut? | To check for possible sensitization/ allergen exposure outside Nunavut |
| Does your child eat the following types of foods (milk, egg, peanut, tree nuts, fish or shellfish) or has eaten them in the past without an allergic reaction? | To get an idea about possible sensitization specifically to those high allergenic foods |
| Any family history of food allergy, environmental allergy, asthma or eczema? | To identify family history of atopy |
| Was your child exclusively breast fed during the first 4 months of life? | To check for possible benefit in decreasing allergy |
| Was your child given the TB vaccination at birth (also called BCG) to prevent a disease called Tuberculosis? | To check for possible benefit in decreasing allergy |
| Was your child ever been hospitalized because of a lung infection or bronchiolitis? | To check for possible effect in regard to Asthma development |
| Number of people (adults and children including this child) living in the house | To calculate crowdedness index |
| Number of bed rooms | To calculate crowdedness index |
