# Supplementary material for: Evaluation of eczema, asthma, allergic rhinitis and allergies among the Grade-1 children of Iqaluit
Source: Allergy Asthma Clin Immunol. 2018 Feb 27;14:9. doi: 10.1186/s13223-018-0232-2 (PMC5827980; doi:10.1186/s13223-018-0232-2)
Supplement: Supplementary file 3 — Additional file 3: Appendix S3. Tested allergens. [file 13223_2018_232_MOESM3_ESM.docx]

Appendix-3

Allergens tested in the study

| **Environmental allergens** | **Food allergens** |
| --- | --- |
| Tree mix | Cow’s milk |
| Grass mix | Egg white |
| Ragweed | Soy |
| Weed | Wheat |
| Mold | Peanut |
| House dust mite | Tree nut mix |
| Cat |  |
| Dog |  |
