# Supplementary material for: Evaluation of eczema, asthma, allergic rhinitis and allergies among the Grade-1 children of Iqaluit
Source: Allergy Asthma Clin Immunol. 2018 Feb 27;14:9. doi: 10.1186/s13223-018-0232-2 (PMC5827980; doi:10.1186/s13223-018-0232-2)
Supplement: Supplementary file 4 — Additional file 4: Appendix S4. Post hoc power analysis findings. [file 13223_2018_232_MOESM4_ESM.docx]

**Appendix-4**

| **Studied associations** | **Power** |
| --- | --- |
| Being ever outside Nunavut and current asthma | 0.397 |
| Being ever outside Nunavut and previous asthma | **0.616** |
| Being ever outside Nunavut and current eczema | 0.058 |
| Being ever outside Nunavut and previous eczema | 0.253 |
| Having a dog and current eczema | **0.634** |
| Having a dog and previous eczema | 0.316 |
| Having a cat and current asthma | 0.131 |
| Having a cat and previous asthma | 0.065 |
| Having a cat and current eczema | 0.086 |
| Having a cat and previous eczema | 0.131 |
| A smoker at home and current asthma | 0.068 |
| A smoker at home and previous asthma | 0.05 |
| A smoker at home and current eczema | 0.055 |
| A smoker at home and previous eczema | 0.059 |
| Family history of eczema and having current eczema | **0.547** |
| Family history of eczema and having previous eczema | **0.734** |
| Family history of asthma and having current asthma | 0.220 |
| Family history of asthma and having previous asthma | **0.713** |
| Exclusive breast feeding till 4 months and current asthma | 0.057 |
| Exclusive breast feeding till 4 months and previous asthma | 0.241 |
| Exclusive breast feeding till 4 months and current eczema | 0.215 |
| Exclusive breast feeding till 4 months and previous eczema | 0.051 |
| Previous respiratory hospitalization and current asthma | 0.09 |
| Previous respiratory hospitalization and previous asthma | 0.10 |
| Being ever outside Nunavut and positive skin test to trees | 0.056 |
| Being ever outside Nunavut and positive skin test to grass | **0.608** |
| Having a cat at home and positive skin test to cat | **0.655** |
| Exclusive breast feeding till 4 months and positive skin test to peanut | 0.175 |
| Exclusive breast feeding till 4 months and positive skin test to egg white | 0.175 |
| Exclusive breast feeding till 4 months and positive skin test to tree nut | 0.31 |
| Previous respiratory hospitalization and positive skin test to trees | 0.485 |
| Previous respiratory hospitalization and positive skin test to grass | 0.10 |

Appendix-4 (Table-6): Post hoc power analysis findings
